# Computational study of transcatheter aortic valve replacement based on patient-specific models—rapid surgical planning for self-expanding valves

**DOI:** 10.3389/fphys.2024.1407215

**Published:** 2024-06-06

**Authors:** Zhuangyuan Meng, Haishan Zhang, Yunhan Cai, Yuan Gao, Changbin Liang, Jun Wang, Xin Chen, Liang Guo, ShengZhang Wang

**Affiliations:** ^1^ Department of Aeronautics and Astronautics, Institute of Biomechanics, Fudan University, Shanghai, China; ^2^ Department of Cardiology, First Hospital of China Medical University, Shenyang, China; ^3^ Department of Anesthesia, First Hospital of China Medical University, Shenyang, China; ^4^ Department of Cardiovascular Ultrasound, First Hospital of China Medical University, Shenyang, China; ^5^ Academy for Engineering and Technology, Institute of Biomedical Engineering Technology, Fudan University, Shanghai, China

**Keywords:** finite element analysis, transcatheter aortic valve replacement, structural simulation, self-expanding valve, computational fluid dynamics

## Abstract

Transcatheter aortic valve replacement (TAVR) is a minimally invasive interventional solution for treating aortic stenosis. The complex post-TAVR complications are associated with the type of valve implanted and the position of the implantation. The study aimed to establish a rapid numerical research method for TAVR to assess the performance differences of self-expanding valves released at various positions. It also aimed to calculate the risks of postoperative paravalvular leak and atrioventricular conduction block, comparing these risks to clinical outcomes to verify the method’s effectiveness and accuracy. Based on medical images, six cases were established, including the aortic wall, native valve and calcification; one with a bicuspid aortic valve and five with tricuspid aortic valves. The parameters for the stent materials used by the patients were customized. High strain in the contact area between the stent and the valve annulus may lead to atrioventricular conduction block. Postoperatively, the self-expanding valve maintained a circular cross-section, reducing the risk of paravalvular leak and demonstrating favorable hemodynamic characteristics, consistent with clinical observations. The outcomes of the six simulations showed no significant difference in valve frame morphology or paravalvular leak risk compared to clinical results, thereby validating the numerical simulation process proposed for quickly selecting valve models and optimal release positions, aiding in TAVR preoperative planning based on patients’geometric characteristics.

## 1 Introduction

Aortic stenosis is one of the most common heart valve diseases, mainly caused by degenerative calcification of the aortic valve or a bicuspid aortic valve with congenital defects (Siu and Silversides, 2010; Pawade et al., 2015). Clinical data research has found that the incidence of severe aortic stenosis is about 3% in people over 75 years old (Lindroos et al., 1993). Aortic stenosis leads to a decrease in cardiac output and may be accompanied by aortic regurgitation, resulting in systemic hypoperfusion, long-term consequences may include cardiac hypertrophy, dilation, and severe heart failure (Kanwar et al., 2018; Pibarot et al., 2019; Spitzer et al., 2019). Transcatheter aortic valve replacement (TAVR) is a minimally invasive surgery developed in recent years for the treatment of aortic stenosis, mainly suitable for patients who are inoperable or at high risk. Compared to traditional surgical aortic valve replacement (SAVR), TAVR has significant advantages such as smaller surgical trauma, faster patient recovery, and shorter postoperative hospital stay (Waksman et al., 2018; Siontis et al., 2019). With the upgrading of TAVR products and the maturity of implantation techniques, the application range of TAVR is gradually expanding. The effectiveness of TAVR in the intermediate-risk patient population has been fully confirmed, showing a trend towards opening up to low-risk patients (Leon et al., 2016; Popma et al., 2019). However, complex postoperative complications are the main difficulty in expanding the eligible population for TAVR. Common postoperative complications of TAVR include coronary artery obstruction, aortic root rupture, atrioventricular block, and paravalvular leak (Bagur et al., 2012; Ribeiro et al., 2013). Contact between the valve stent and the aortic wall interferes with atrioventricular conduction, requiring adjustment with a pacemaker postoperatively. The presence of paravalvular leak after TAVR is associated with higher late mortality, cardiovascular mortality, and need for reintervention, and may be related to the deposition and formation of thrombi, thereby increasing the risk of postoperative stroke (Maisano et al., 2015; Bianchi et al., 2019).

Numerical simulation is an effective approach to study TAVR procedures, which can be used to investigate the relationship between pathological morphology, procedural selection, and the effects on valve implantation outcomes. This includes the relationship between calcification distribution and TAVR results, as well as the impact of stent deployment location and height on surgical outcomes, and the prediction of postoperative complications (Morganti et al., 2014; Morganti et al., 2016; Basri et al., 2020). Tzamtzis et al. utilized finite element analysis to study the mechanisms of radial support provided by two types of valves, pointing out the correlation between radial stent support and postoperative stent displacement and atrioventricular conduction block (Tzamtzis et al., 2013). Bianchi et al., based on three clinical cases, combined structural simulation with computational fluid dynamics to study the impact of stent type and implantation depth on postoperative paravalvular leak, demonstrating effective reduction in paravalvular leak post-surgery, consistent with clinical results (Bianchi et al., 2019).

This study aimed to develop a set of numerical simulation methods for rapidly predicting post-TAVR outcomes and potential complications, and to compare the postoperative results of self-expanding valves at different implantation positions, contrasting them with clinical outcomes. In this study, six patient-specific aortic models were established, and a complete transcatheter self-expandable valve implantation process and computational fluid dynamics simulations were constructed to analyze stent deployment morphology, apposition, atrioventricular conduction block, and postoperative paravalvular leak conditions. The numerical simulation results were used to determine the optimal preoperative valve implantation position and valve selection, providing guidance for preoperative planning in TAVR treatment.

## 2 Methods

### 2.1 Case data

In this study, CTA image data of six patients diagnosed with mild calcific aortic stenosis were retrospectively analyzed and chosen for model reconstruction. Of these, one with a bicuspid aortic valve and five with tricuspid aortic valves. This study obtained patient consent, adhering to the Helsinki Declaration, and received approval from the Ethics Committee of the First Hospital of China Medical University. All six patients experienced minor paravalvular leakage post-surgery, and none had a pacemaker implanted.

### 2.2 Aortic root model

**FIGURE 1:**
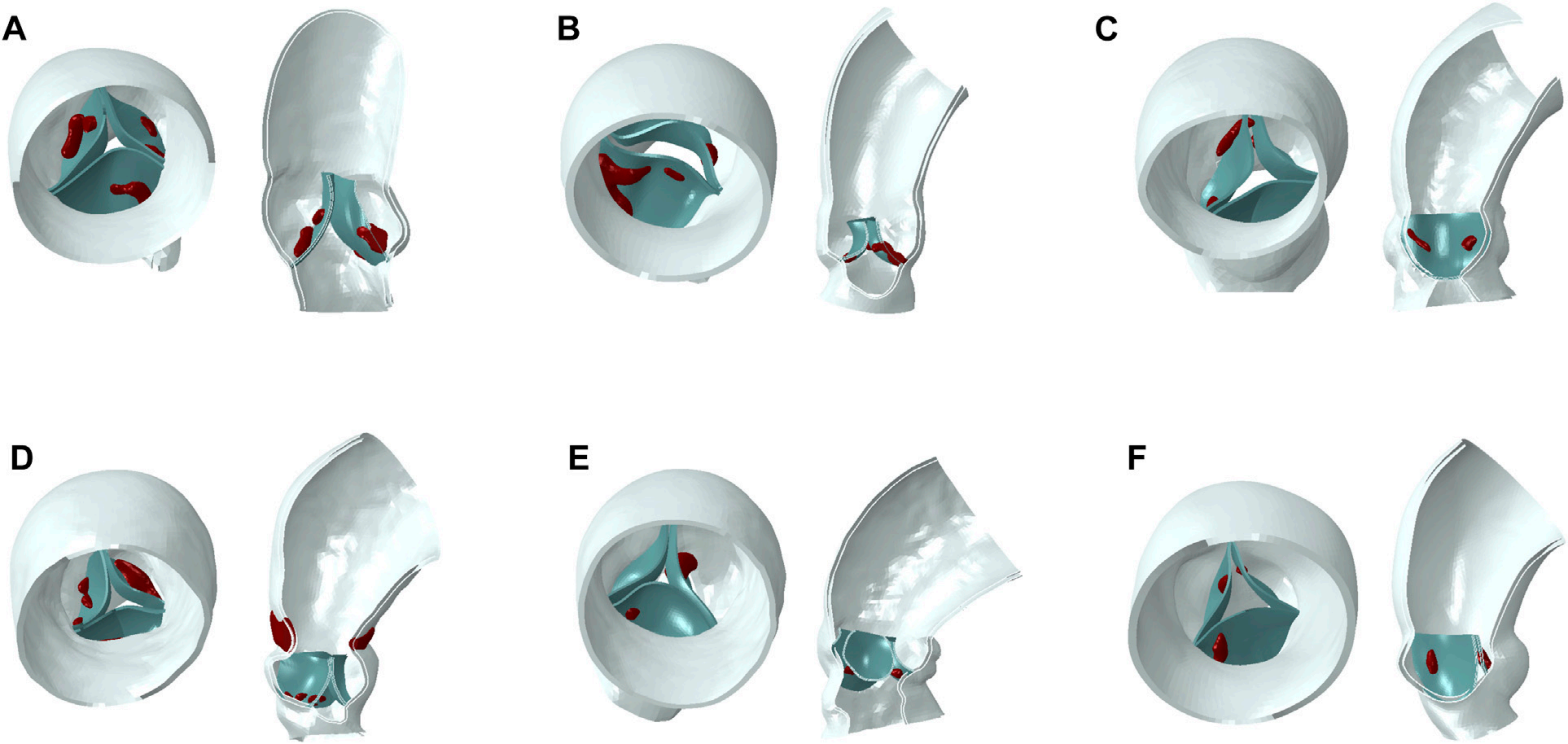
Aortic models (blue part for aortic wall, green part for native valve, red part for calcification). **(A–F)**: model 1 to model 6.

Patient-specific aortic models were reconstructed in MIMICS 20.0 (Materialise, Leuven, Belgium) from the left ventricular outflow tract to the ascending aorta, including the aortic wall, native valve, and calcification. The models were repaired and smoothed in Geomagic Studio 2013 (Geomagic Inc. United States) to generate a standard geometric format. The reconstruction of the native valve was accomplished by selecting control points in Solidworks (Dassault System, Concord, Massachusetts, United States), as shown in Figure 1.

#### 2.2.1 Native valve model

The rapid method for constructing native leaflets involves selecting a specific moment of the native leaflet, then using a fourth-order spline curve to reconstruct a curve of the free edge. The attached edge curve is extracted on the aortic wall and a surface-type native leaflet is constructed in the CAD software Solidworks.

**FIGURE 2:**
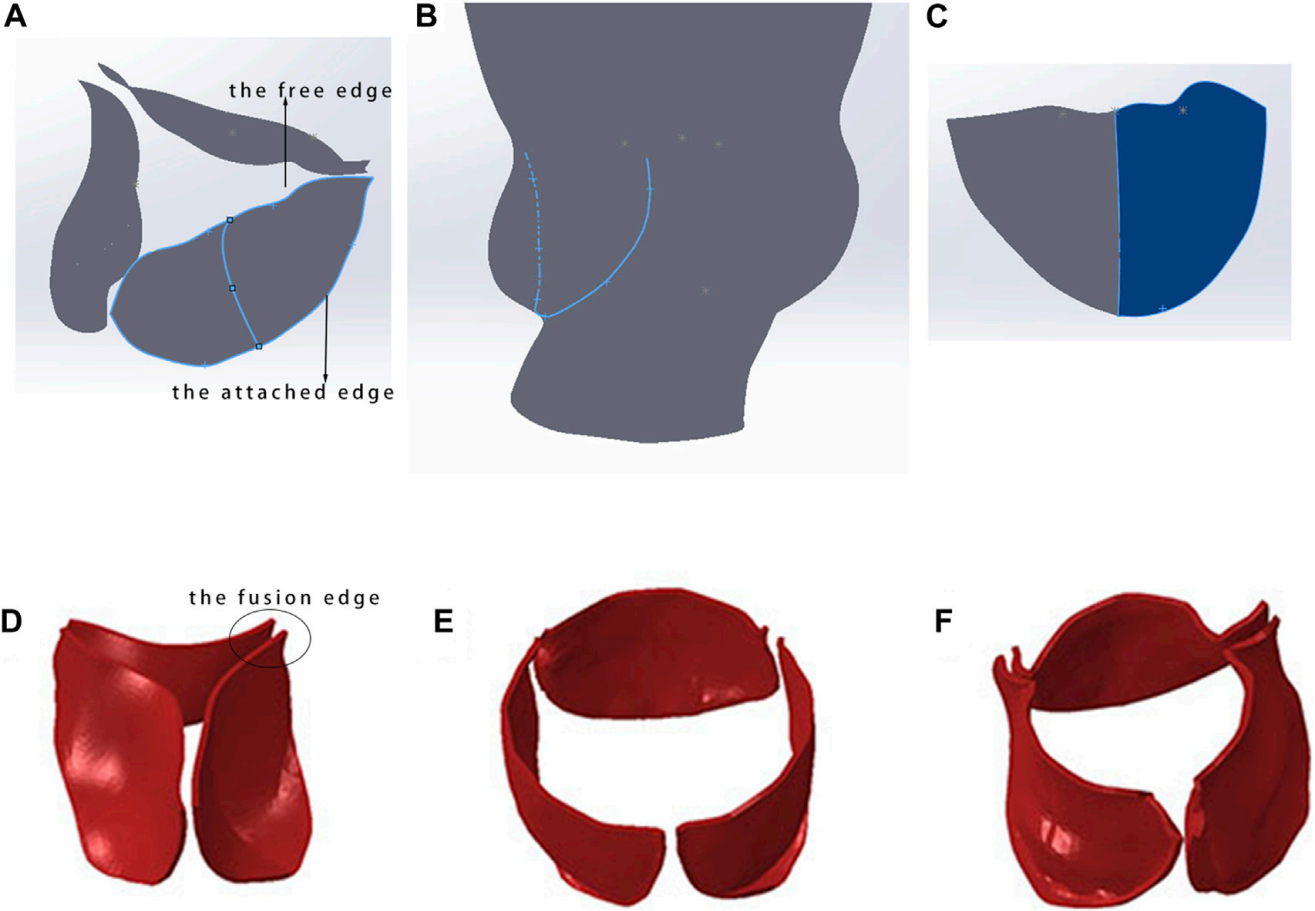
Rapid modeling method for native valve. **(A)** Constructed native valve model; **(B)** extraction of the attachment edge curve; **(C)** spline surface construction of a single leaflet model; **(D)** initial state of the tricuspid native valve; **(E)** morphology of the native valve post-TAVR with a fusion length of 0 mm; **(F)** morphology of the native valve post-TAVR with a fusion length of 3–5 mm.

The native valve of a tricuspid aortic valve typically comprises three leaflets, with the structure and morphology controlled by the attached edge and the free edge of their profiles, as shown in Figure 2A. The rapid modeling process for the native valve involves the following steps: (1) Draw the attached edge curve on the aortic model, as shown in Figure 2B. Sketch the curve of the free edge on the solid native valve, establish the central reference plane of the leaflet through the attachment line and the central point of the free edge, and utilize a third-order spline curve to draw the centerline on this reference plane to regulate the curvature of the leaflet. (2) Utilize the lofted surface feature with the centerline as the control curve to construct the spline surface between the attached edge and the free edge, thereby generating the model of a single leaflet, as shown in Figure 2C. This model allows for rapid adjustment of the free edge and curvature of the leaflet, enabling parametric control of the leaflet modeling. The aorta, calcification, and native leaflets are all modeled within an absolute coordinate system. Ultimately, the assembly of the three models is completed, with the attached edge of the native leaflet bound to the inner surface of the aorta.

The native valve of a bicuspid aortic valve generally consists of two leaflets, and its construction method is similar to that of the tricuspid aortic valve. The difference lies in the greater challenge of determining the endpoints of the free edge and the position of the annulus for the bicuspid valve. This process requires careful identification and accumulated experience in using CT.

#### 2.2.2 Length of leaflet fusion edge

The accuracy of finite element simulation results is closely related to the establishment of the model, especially in dealing with the lengths of fusion ridges and calcification parts of native valve, which require precise modeling based on imaging data. In selecting the fusion ridge length, it is necessary to collaborate with personnel experienced in CT to estimate the approximate fusion length and then perform binding processing in Abaqus (Dassault Systèmes, France). Figures 2D–F shows the influence of fusion ridge length on the final shape of the simulated valve stent.

In this study, all models were meshed in Hypermesh 2019 (Altair, Troy, Michigan, United States). The calcification was meshed with tetrahedral elements. The aortic wall and native valve were meshed with triangles on inner surface with total thickness of 1.5 mm and 0.6 mm. The mesh independence test ensured that the differences in aortic displacement in the model calculations were within 5% (Li et al., 2022). In the process of TAVR simulation, aortic model can be assumed as linear elastic material (Bailey et al., 2016; Luraghi et al., 2020) to reduce the computational cost, and detailed material parameters were listed in Table 1.

**TABLE 1:** Material parameters for aortic model.

Component	Elastic Modulus (MPa)	Poisson’s ratio
Aortic wall	2	0.45
Leaflets	3.3	0.45
Calcification	12.6	0.3

### 2.3 Self-expandable valve model

**FIGURE 3:**
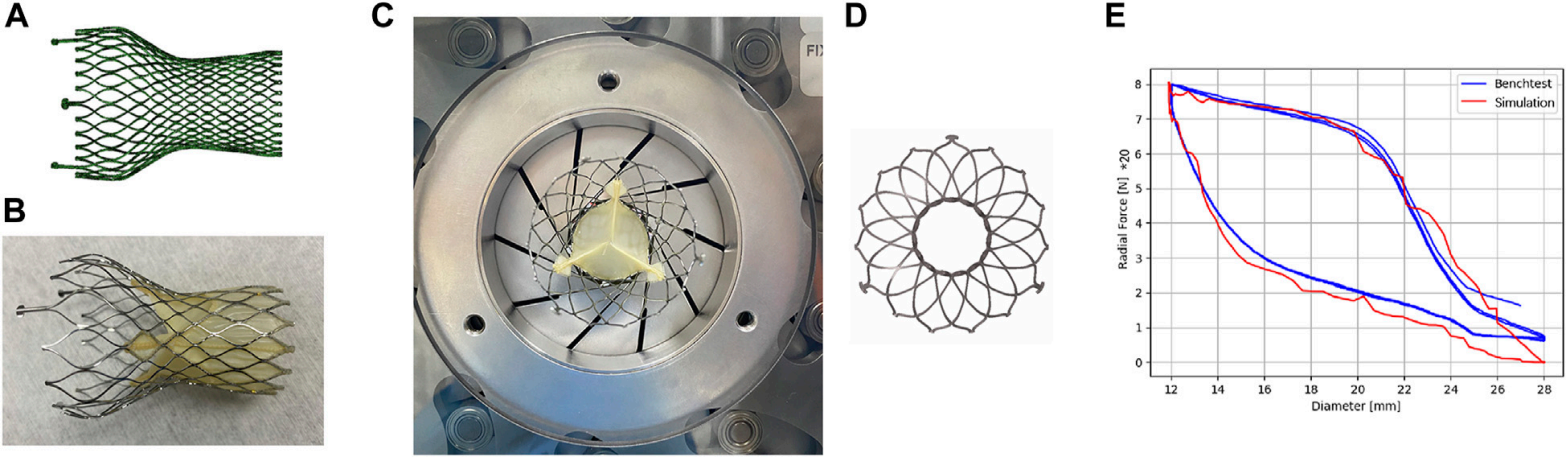
Self-expanding valves. **(A)** The numerical model of Venus A-Valve; **(B)** Venus A-Valve product; **(C)** the Blockwise Crimper system; **(D)** the numerical model of radial force experiment; **(E)** parameter fitting of the stent’s nickel-titanium alloy.

The valve stents implanted for all six patients were Venus A-Valve (Venus MedTech, China). In the simulation, the circumference of native annulus were measured from CT images, and the corresponding diameters were calculated. Therefore, according to the selection principles of Venus A-Valve, a 26 mm size self-expandable valve was chosen for implantation simulation for five cases, while one patient chose a valve stent with a 23 mm size. The geometric model of the Venus A-Valve was established using Solidworks and Abaqus, as shown in Figure 3A. Figure 3B shows the Venus A-Valve product.

In an environment with a temperature range of 37°C ± 2°C, we conducted a radial force experiment using the Blockwise Crimper system (Blockwise Engineering LLC, AZ, United States) to infer the material parameters of the nitinol alloy in the Venus A-Valve, as shown in Figure 3C. The radial force testing device of the valve stent includes a control system and a crimping device, which consists of 10 crimping plates. The crimping plates radially contract to compress the stent and generate reaction force, thereby measuring the stent’s radial force.

The main steps of the stent’s radial force testing are as follows: (1) Place the stent test area inside the crimping head, adjust the initial diameter of the crimping head slightly larger than the outer diameter of the stent, set the stent compression radius (the crimping head movement distance), and start the test; (2) Radially shrink the crimping head, measure the force acting on the crimping plate by the force sensor, and obtain the curve of the stent’s radial force with the change in diameter. In this study, the metal crimping steel plate compressed the stent from a diameter of 28 mm–12 mm at a speed of 0.25 mm/s, then released it back to its original diameter, repeating this process three times. Figure 3D shows the numerical model of radial force experiment.

In the simulation model, a rigid circular tube was used to radially compress the stent, extracting the normal contact force acting on each unit of the compressed circular tube and summing them to obtain the radial force of the stent in its current state. By setting the output results at multiple time points, the relationship curve between the stent diameter and radial force was obtained. The results of the radial force testing and simulation of the valve stent are shown in Figure 3E. From the results in the figure, it can be seen that the radial support force tested when compressed to a diameter of 12 mm was 160 N, while the simulation result was 158.5 N, with an error of 0.9%. Additionally, the nitinol alloy material exhibited good matching of the loading phase transition point and unloading phase transition point. The simulation model of the stent and the radial force with diameter variation in both testing and simulation show a high degree of consistency in peak value, trend, and inflection point, validating the accuracy of the stent’s structural and material modeling and the material parameters of the nitinol alloy in the Venus A-Valve were listed in Table 2.

**TABLE 2:** Material parameters for self-expandable stent.

Parameter	Description	Value
$E_{A}$	Austenite elastic modulus	55,000 MPa
$\nu_{A}$	Austenite Poisson’s ratio	0.33
$E_{M}$	Martensite elastic modulus	30,000 MPa
$\nu_{M}$	Martensite Poisson’s ratio	0.33
$\varepsilon^{L}$	Transformation strain	0.045
$\sigma_{L}^{s}$	Start of transformation loading	260 MPa
$\sigma_{L}^{E}$	End of transformation loading	550 MPa
$\sigma_{U}^{s}$	Start of transformation unloading	80 MPa
$\sigma_{U}^{E}$	End of transformation unloading	30 MPa
ρ	Material density	6,300 kg/m^3^

Previous studies have shown that if the focus of the research is on the dynamic behavior of the stent during simulations and its forces acting on the vascular, or to reduce computational costs, beam elements can be used (Luraghi et al., 2022). In this study, the stent mesh was created using B31 beam elements, and based on mesh sensitivity tests, a mesh size of 0.02 mm was chosen for the stent connection point, while the rest of the stent was meshed with a size of 0.1 mm, the number of elements of stent was about 89168.

### 2.4 TAVR simulation

The simulation of TAVR process was performed in Abaqus. The implantation of the TAVR stent induces remodeling of the aorta, constraints are applied to both ends of the aorta to limit its axial movement. From a physiological standpoint, zones of calcification develop due to the abnormal differentiation of cells in the valvular leaflets (Fisher et al., 2013), which are integral to the structure of the valve. Consequently, the calcification becomes adherent to the native valve. Studies have shown that the skirt and prosthetic valve have less than a 3% impact on the outcome of stent deployment (Bailey et al., 2016); therefore, the skirt and prosthetic valve are not considered in order to avoid complex soft material contact, reduce model complexity, and improve computational efficiency.

**FIGURE 4:**
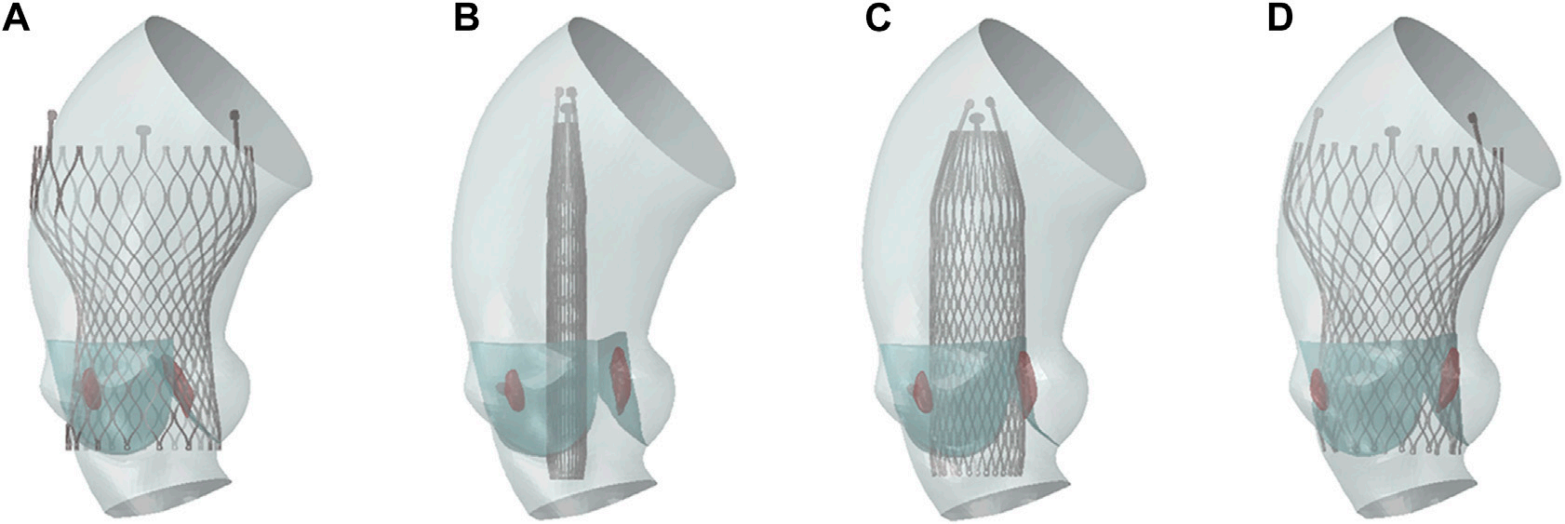
TAVR implantation process. **(A)** Initial state; **(B)** stent crimping; **(C)** stent expanding; **(D)** stent releasing.

Nitinol can autonomously return to its original shape after the constraints are removed, and the valve implantation process is generally completed with crimping and releasing steps (Morganti et al., 2016; Nappi et al., 2021). In this study, the complete TAVR process in clinical operation was restored. The complete self-expandable valve implantation process established was shown in Figure 4. The TAVR simulation process involved the following three steps:

1. Stent crimping: A rigid cylindrical tube and the prosthetic valve were assembled concentrically and adjusted to the appropriate implantation height. A radially inward displacement boundary condition was applied to the cylindrical tube surface, causing it to shrink radially to a diameter of 9 mm. Additionally, a uniform pressure of 0.15 MPa was applied to the ventricular side surface of the native valve to ensure it was in an open state, making certain that there was no initial mesh interference between the stent and the native valve. To ensure stability, a general contact configuration was established between the stent and the rigid cylindrical surface, with a contact friction coefficient set at 0.15 to inhibit any relative sliding among the components. The radius of the valve was reduced under the driving of the rigid cylinder to obtain the crimped state.
2. Stent expanding: In this step, a radial outward displacement condition was applied to the rigid cylindrical tube to increase its diameter. Due to the superelasticity of the material, the self-expandable stent gradually recovered its shape and came into contact with the native valve and aortic wall. In these first two steps, the entire TAVR device and the aortic model are considered to be mutually independent, with no contact defined between them.
3. Stent releasing: The self-expandable valve slightly contracted under the elastic recovery force of the aortic root and native valve, and eventually the whole model reached a final balance.

### 2.5 Calculation of paravalvular leak

Computational fluid dynamics (CFD) was used for post-operative paravalvular leak analysis. Due to the tiny penetrations or gaps in the models after valve implantation, it was difficult to construct an effective and stable computational mesh using traditional CFD methods, such as finite volume method. Therefore, the Lattice Boltzmann Method (LBM) was employed to calculate the regurgitation caused by paravalvular leak. LBM is a meshless method, which is easy to match with complex geometric boundaries. Regarding the thin valve leaflet boundaries, the LBM possesses the flexibility to adapt the boundary treatment at lattice nodes. This adaptability is essential for accurately simulating the fluid dynamics behavior around thin boundaries. The calculation was completed in the software XFlow 2019 (Dassault System, France).

After the valve implantation, the morphologies of the aorta and the stent, post deformation, were obtained. The deformation model results from Abaqus were exported in STL format. This study aims to analyze the regurgitant flow around the valve, which primarily occurs during diastole. This happens when blood flows back through the small gaps around the valve frame after it is completely closed. Therefore, it is crucial to ensure that the prosthetic valve is in a fully closed state during diastole for the analysis, to eliminate the impact of blood regurgitation back into the valve. In Geomagic, the prosthetic valve were adjusted to a fully closed state. The deformation results of the aorta, native valve, stent, and the closed prosthetic valve model were then imported into the XFlow software for assembly. Through mesh convergence analysis using regurgitation flow as the inspection index, the global lattice size was finally determined to be 0.25 mm, with local refinement to 0.1 mm on the stent wall and native valve. Paravalvular leak primarily occurs during diastole, when blood flows to the ventricle side from the aorta. The calculation was considered as steady flow, with average diastolic aortic pressure of 10000 Pa applied for inlet condition in ascending aorta side and diastolic left ventricular pressure of 2000 Pa applying for outlet in ventricle side. Blood was assumed as Newtonian fluid with density of 1,060 kg/m^3 and kinematic viscosity of 0.0035 Pa∙s, the cardiac cycle was assumed as 0.8 s.

The convergence criterion for the calculation is based on the flow rate at the outlet, where convergence is achieved when the variation in flow rate is less than 0.1%. The calculated flow rate at the ventricular outlet, attributed to paravalvular leak, is then used to determine the severity of the leak according to clinical evaluation standards.

## 3 Results

According to the Venus A-Valve release position standard, the depths of the stents released are 0 mm, 2-3 mm, and 5 mm below the original valve annulus, corresponding to zero position, high position, and standard position, respectively. Establish three different implantation depth models to analyze the impact of varying implantation depths on the outcomes of TAVR.

### 3.1 Aortic stress distribution

**FIGURE 5:**
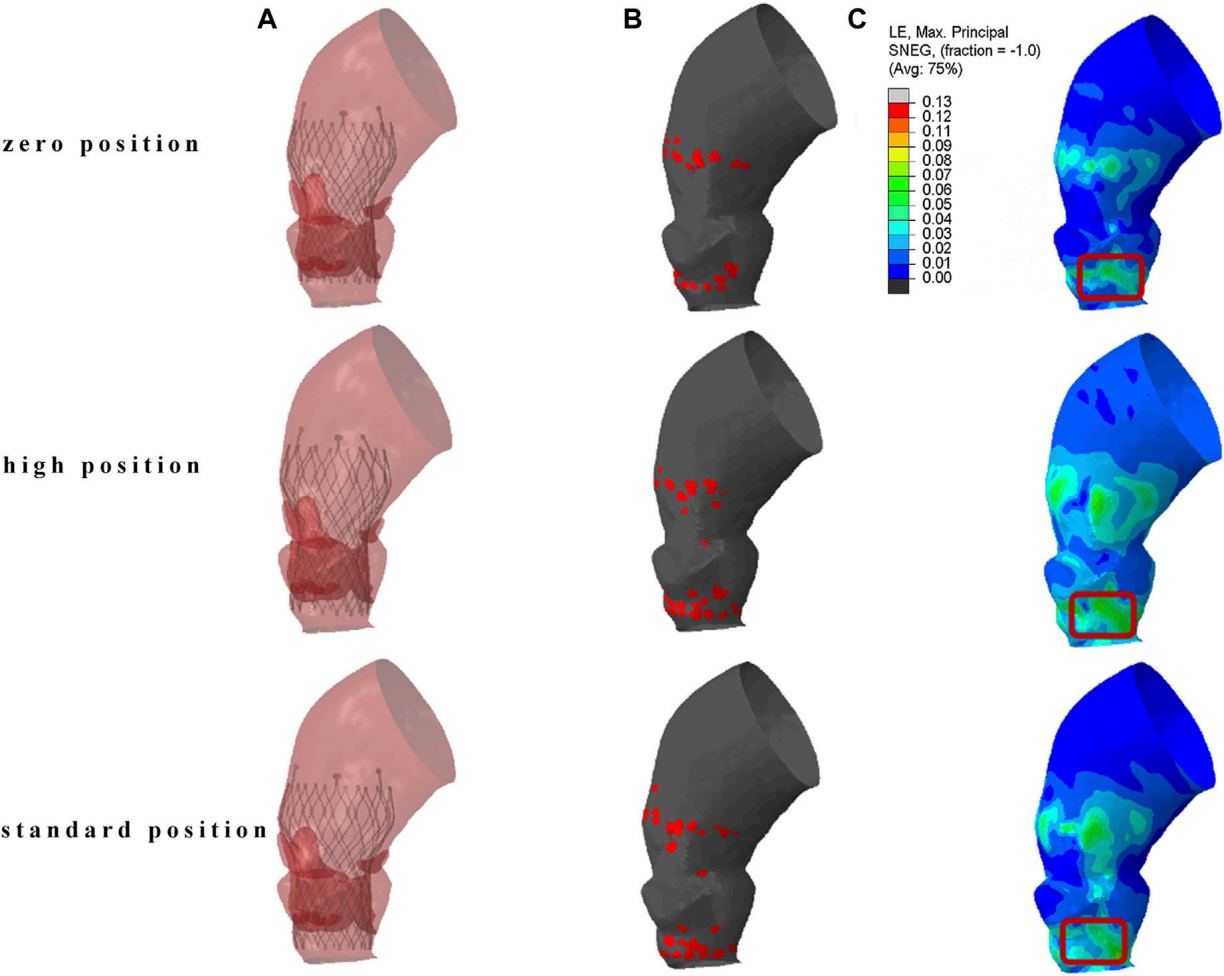
The result of TAVR implantation at different positions. **(A)** Finite element simulation results; **(B)** contact area between the stent and the inner surface of the aorta; **(C)** maximum principal logarithmic strain of the aorta.

The results of TAVR implantation at the zero position, high position, and standard position in Model 4 are shown in Figure 5. Figure 5A shows the finite element simulation results, and Figure 5B shows the contact area between the stent and the inner surface of the aorta. The maximum principal strain distribution of the aorta is shown in Figure 5C. The high strain regions are mainly concentrated in the area of the valve annulus and the sinotubular junction. There are also obvious circular high strain areas distributed along the circumferential direction below the valve annulus, which are generated due to the contact between the end of the stent and the left ventricular outflow tract. The peak stresses occur at the connection between the aortic root sinus and the native valve, with values of 0.211 MPa, 0.195 MPa, and 0.182 MPa, respectively.

The localized stresses exerted by the device frame on the membranous septum (MS), which is located between the aortic annulus and the bundle of His, as shown in the red marked area in Figure 5C, may disrupt cardiac conduction and lead to resultant cardiac conduction abnormalities (CCA). The percentage of this area, known as the contact pressure index (CPI), and the maximum contact pressure (CPMax) exerted were recorded. In Model 4, the CPMax value was 0.140 MPa. When the reference is set at 0.4 MPa, the CPI is 0, this indicates that the pressure exerted by the implanted valve stent on the membranous septum is below the threshold that could potentially alter the cardiac conduction system.

### 3.2 Deformation of the valve stent

**FIGURE 6:**
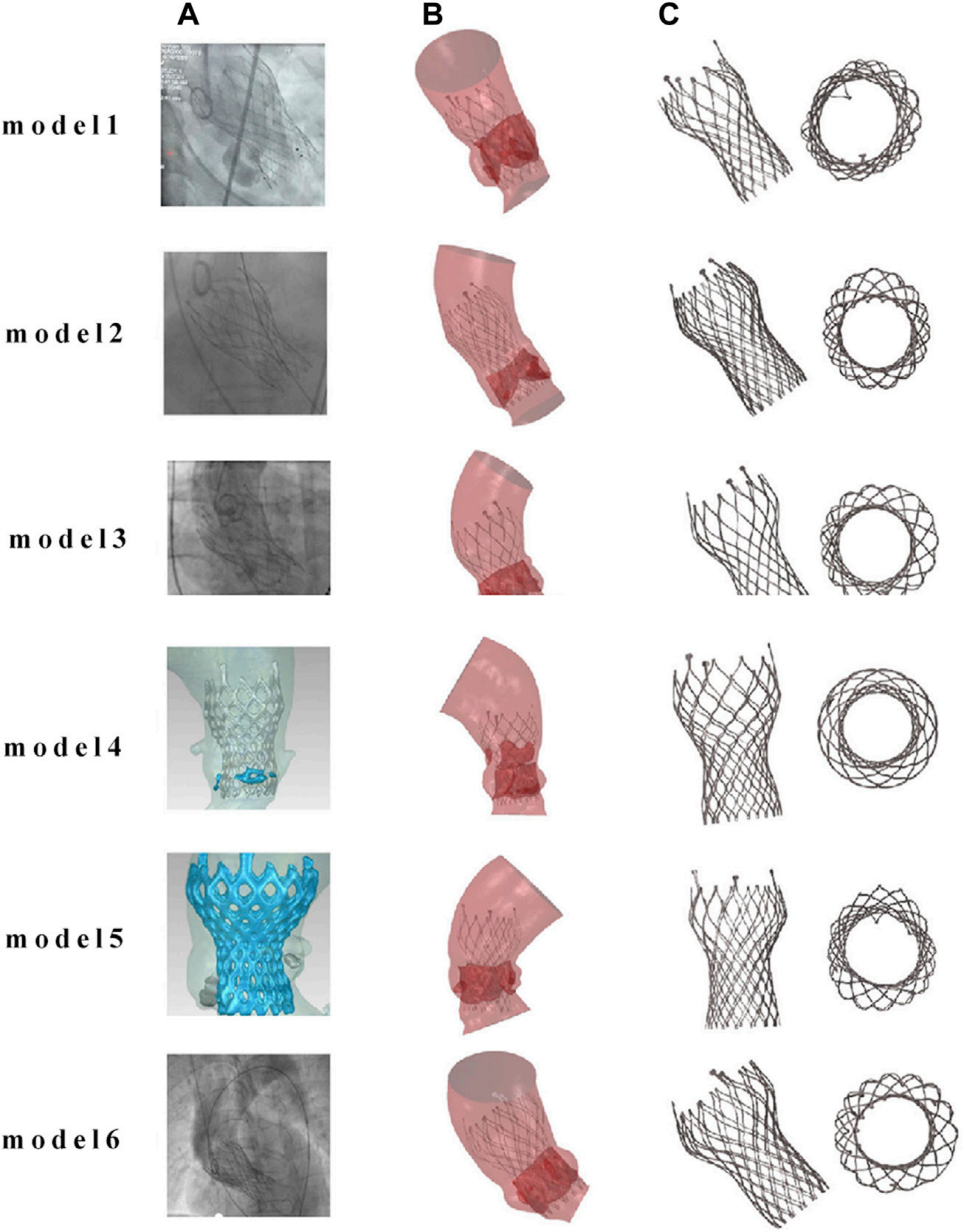
Comparison of finite element simulation results with angiography for the same implantation position. **(A)** Clinical images of TAVR positioning after implantation; **(B)** finite element simulation images after TAVR implantation; **(C)** post implantation morphology of the valve frame.

The post-implantation morphology of the valve stent is a representation of the functional and durable characteristics of the implanted prosthetic valve. In clinical practice, the valve stent release positions for Model 1 and Model 4 are designated as the standard position, while for the remaining patients, it is at the zero position release, as shown in Figure 6.

**FIGURE 7:**
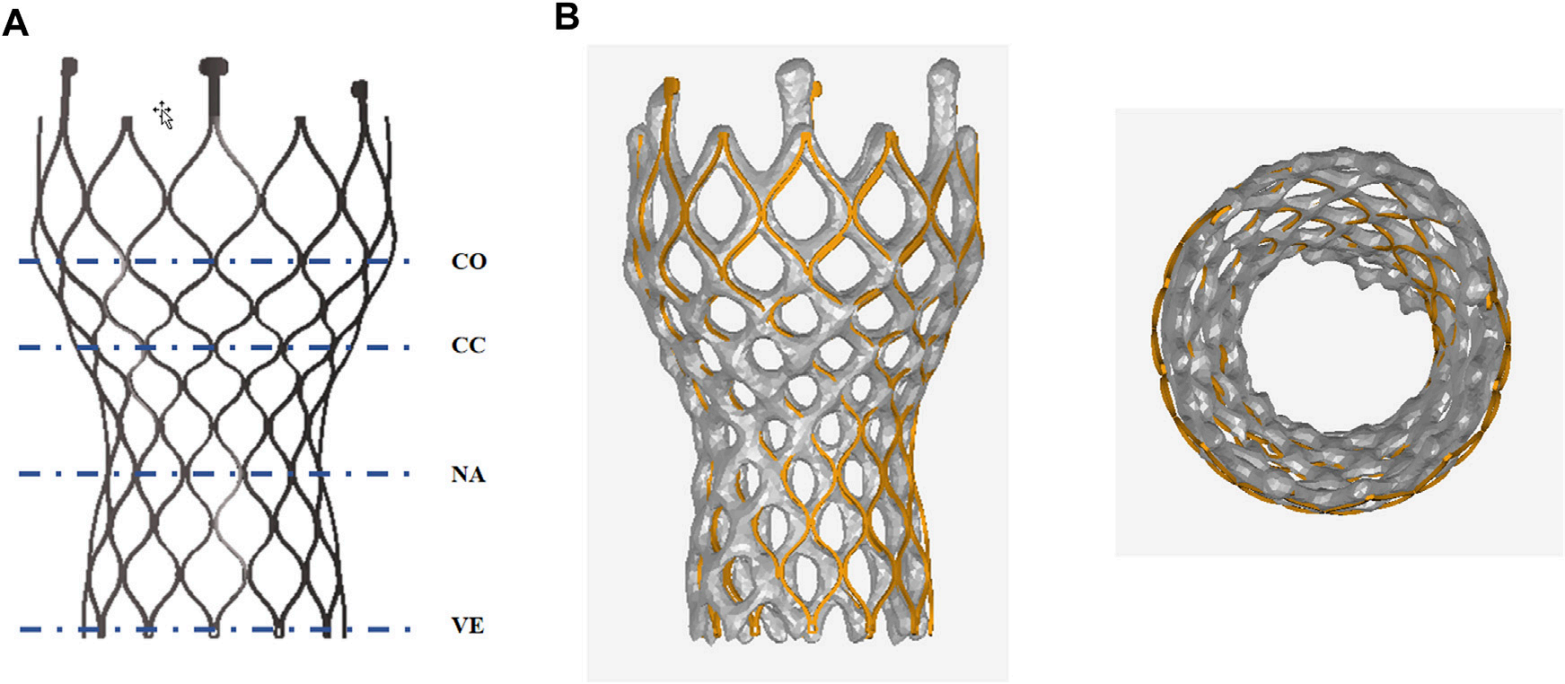
Anatomy after the valve stent implantation. **(A)** Commissures (co), central coaptation (cc), nadir (na), and ventricular end (ve); **(B)** Comparison of finite element simulation results with clinical outcomes post-valve implantation.

Figure 7 compares the morphology of the valve stent obtained from post-implantation CT scans in clinical practice with the results obtained from finite element simulations. Quantitative comparisons were conducted on four sections of the implanted valve stent: commissures, central coaptation, nadir, and ventricular end, calculating their diameters, perimeters, and areas to verify the accuracy of the differences between the clinical post-implantation stent deformation and simulated stent deformation. For Model 4, the clinical post-implantation maximum diameters are 32.28 mm, 21.40 mm, 21.25 mm, and 22.98 mm for these sections, respectively, compared to simulation results of 32.19 mm, 22.13 mm, 21.26 mm, and 22.96 mm. The corresponding error percentages are 0.28%, 3.98%, 0.05%, and 0.09%, respectively. Similar analyses of the minimum diameters, perimeters, and areas revealed errors of 0.40%, 2.27%, 0.14%, and 1.26% for minimum diameters; 0.11%, 1.20%, 0.33%, and 0.71% for perimeters; and 0.12%, 1.43%, 0.27%, and 0.80% for areas, respectively.

### 3.3 Analysis of paravalvular leak

**FIGURE 8:**
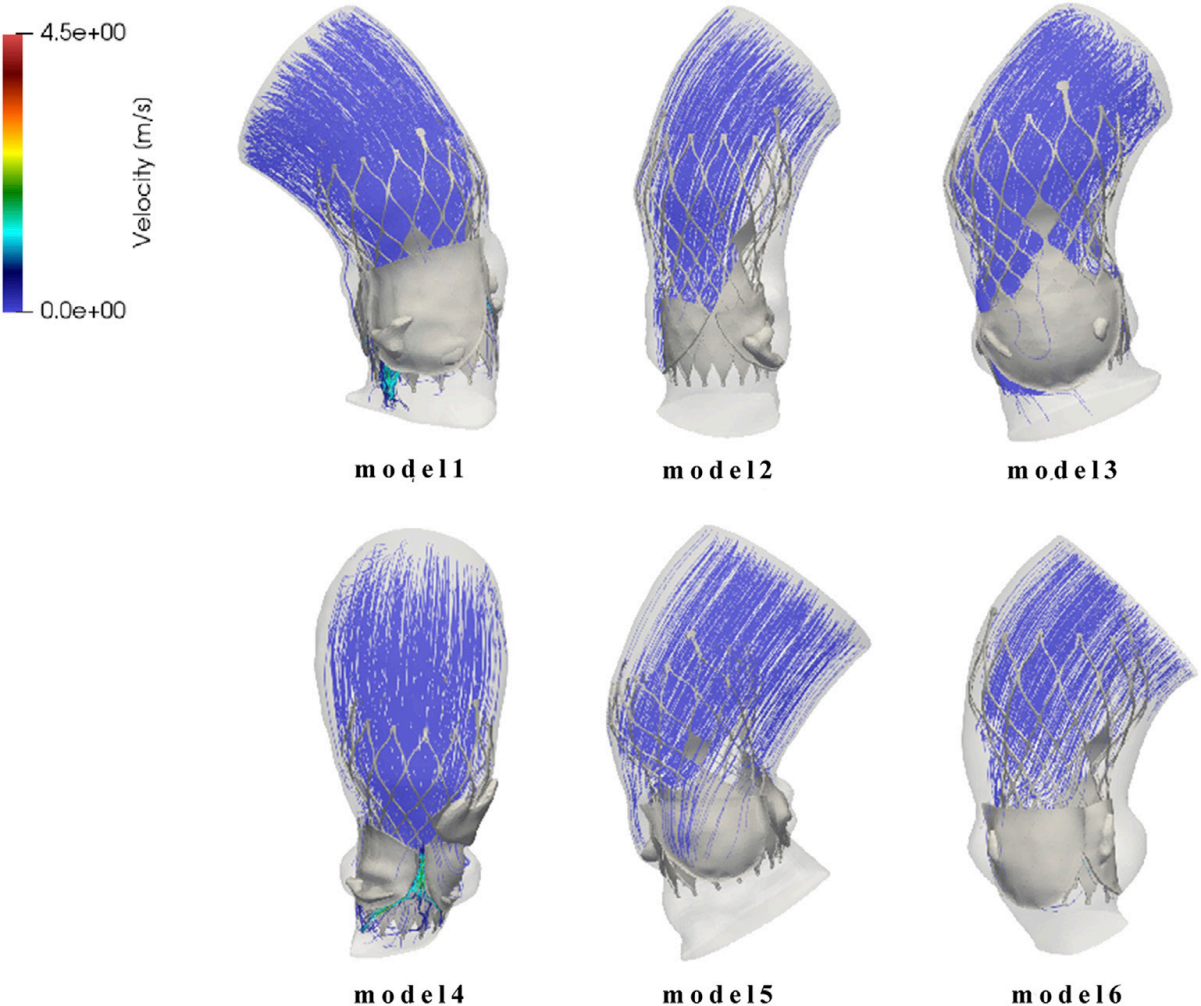
Flow field distribution and streamline diagram after prosthetic valve implantation for model 1 to model 6.

Since the calcified valve could not fully expand, the stent was blocked by the apposition area of the leaflets during releasing, so that it could not fit well to the wall at the commissure edge, forming a significant gap area, as shown in Figure 8. It shows the location of paravalvular leak in each model for the six cases. The occurrence of paravalvular leak was mainly concentrated at the commissure edge of the native valve, which was consistent with the location of the gap formed by stent inadequate apposition. The regurgitation flow rates for Model 1 to Model 6 are 1.0 mL/s, 0.6 mL/s, 1.6 mL/s, 8.0 mL/s, 3.9 mL/s, and 2.9 mL/s respectively, indicating mild paravalvular leakage consistent with clinical results.

## 4 Discussion

This study established a simulation process for TAVR using self-expanding valves, which is based on patient-specific aortic models. It analyzed aortic stress, valve frame morphology, atrioventricular conduction block, and paravalvular leaks after valve stent implantation, and compared these factors with clinical postoperative outcomes.

The study found that the valve frame can generate high strain on the aortic wall. High aortic stress favors the fixation of the valve frame, but it also increases the risk of aortic rupture. Additionally, high strain may cause damage and embolization of calcified tissue on the aortic valve, leading to stroke (Nombela-Franco et al., 2012; Van Mieghem et al., 2015). Previous research has shown a correlation between embolized calcified tissue and postoperative stroke. The lower stress exerted by self-expanding stents on the aorta can decrease the risk of postoperative stroke, consistent with clinical findings of lower post-implantation stroke rates with CoreValve (Biondi-Zoccai et al., 2014).

The post-implantation morphology of the valve frame can measure the functionality of the prosthetic valve and its durability. It is generally believed that a more circular valve frame morphology in the suture area is more advantageous for the normal function and longevity of the prosthetic valve, whereas a non-circular frame shape may affect the valve’s morphology during opening and closing, causing folding and distortion of the prosthetic valve leaflets (Morganti et al., 2014; Nappi et al., 2021). The valve frame’s radial support has a stronger reshaping effect on the aorta post-implantation, helping to maintain a circular cross-sectional shape. The paravalvular gap may be a potential site for thrombus formation (Bianchi et al., 2019), with the severity of paravalvular leak correlating with the long-term survival rate of patients (Maisano et al., 2015). The study results show better wall apposition with smaller paravalvular gap areas in all cases post valve implantation, indicating a lower risk and severity of postoperative paravalvular leak, consistent with clinical observations (Biondi-Zoccai et al., 2014; Gonska et al., 2017).

There are limitations in the computational model due to simplifications made for modeling purposes. The simulation did not consider the skirt and prosthetic valve leaflets during the valve frame crimping process, leading to a potential underestimation of the crimping degree, which could introduce significant errors in fatigue analysis but was not within the scope of this study. The simulation of TAVR valve implantation also did not account for the prosthetic valve leaflets and skirt, although previous research suggests that this approach has no significant impact on TAVR frame placement results (Bailey et al., 2016).

The study only used six aortic models, all with mild calcification, and some cases only had calcification on the aortic side of the native leaflets, without direct contact with the valve frame. This modeling assumption is consistent with clinical findings and other studies on the impact of calcification patterns on TAVR outcomes (Yip and Simmons, 2011; Sturla et al., 2016; Luraghi et al., 2020). In reality, calcification often occurs on the ventricular side of the aortic valve. In such cases, the harder calcified area will have direct contact with the frame, posing a stronger hindrance to frame implantation and potentially leading to more severe frame deformation and distortion. The study did not delve deeper into this aspect of calcification.

## 5 Conclusion

This study established a complete simulation process using patient-specific models to predict outcomes of TAVR in different positions, results were compared to clinical outcomes. (1) Using the Venus A valve stent offered greater post-implantation support and reduced perivalvular leakage risk. However, this also increased aortic stress leading to potential conduction pathway damage and the need for pacemaker implantation. Placing the valve in zero or high positions reduced this risk. (2) A numerical evaluation method was developed to aid in selecting optimal valve models and release positions for TAVR preoperative planning based on patient-specific characteristics.

## Data Availability

The original contributions presented in the study are included in the article/Supplementary material, further inquiries can be directed to the corresponding authors.
