# Corrigendum: Computational study of transcatheter aortic valve replacement based on patient-specific models—rapid surgical planning for self-expanding valves

**DOI:** 10.3389/fphys.2025.1541483

**Published:** 2025-01-22

**Authors:** Zhuangyuan Meng, Haishan Zhang, Yunhan Cai, Yuan Gao, Changbin Liang, Jun Wang, Xin Chen, Liang Guo, ShengZhang Wang

**Affiliations:** ^1^ Department of Aeronautics and Astronautics, Institute of Biomechanics, Fudan University, Shanghai, China; ^2^ Department of Cardiology, First Hospital of China Medical University, Shenyang, China; ^3^ Department of Anesthesia, First Hospital of China Medical University, Shenyang, China; ^4^ Department of Cardiovascular Ultrasound, First Hospital of China Medical University, Shenyang, China; ^5^ Academy for Engineering and Technology, Institute of Biomedical Engineering Technology, Fudan University, Shanghai, China

**Keywords:** finite element analysis, transcatheter aortic valve replacement, structural simulation, self-expanding valve, computational fluid dynamics

In the published article, there was an error in Table 2 as published. It does not match the data included in our initial submission. The corrected Table 2 and its caption appear below.

**TABLE 2:** Material parameters for self-expandable stent.

Parameter	Description	Value
$E_{A}$	Austenite elastic modulus	55,000 MPa
$\nu_{A}$	Austenite Poisson’s ratio	0.33
$E_{M}$	Martensite elastic modulus	30,000 MPa
$\nu_{M}$	Martensite Poisson’s ratio	0.33
$\varepsilon^{L}$	Transformation strain	0.045
$\sigma_{L}^{s}$	Start of transformation loading	260 MPa
$\sigma_{L}^{E}$	End of transformation loading	550 MPa
$\sigma_{U}^{s}$	Start of transformation unloading	80 MPa
$\sigma_{U}^{E}$	End of transformation unloading	30 MPa
ρ	Material density	6,300 kg/m^3^

The authors apologize for this error and state that this does not change the scientific conclusions of the article in any way. The original article has been updated.

